# Transient Serotonin Toxicity Evoked by Combination of Electroconvulsive Therapy and Fluoxetine

**DOI:** 10.1155/2014/162502

**Published:** 2014-06-01

**Authors:** René Klysner, Birgitte Bjerg Bendsen, Maja Soon Hansen

**Affiliations:** Psychiatric Centre Frederiksberg, Nordre Fasanvej 57-59, 2000 Frederiksberg, Denmark

## Abstract

The serotonin syndrome has been described only in rare instances for electroconvulsive therapy combined with an antidepressant medication. We describe a case of serotonin toxicity induced by electroconvulsive therapy in combination with fluoxetine.

## 1. Introduction

Drugs that enhance serotonin neurotransmission can induce the serotonin syndrome in animals and patients [1, 2]. The symptoms in patients as originally described by Sternbach in 1991 were mental status changes, agitation, myoclonus, hyperreflexia, diaphoresis, shivering, tremor, diarrhea, incoordination, and fever [2].

The serotonin syndrome has been described with monotherapy but most often with combinations of drugs. Okamoto et al. [3] described a case of serotonin syndrome by concurrent use of electroconvulsive therapy (ECT) and paroxetine. They state that there were hardly any reports of induction of serotonin syndrome by combination of ECT with antidepressants.

We present the case of a female patient with major depressive disorder who developed serotonin toxicity during a combination of ECT and fluoxetine treatment.

## 2. Case Presentation

A 31-year-old woman had no past history of physical disease except for asthma for which she received daily inhalations with budesonide and formoterol. She had recurrent depression and had been treated over some years with citalopram, dosulepin, mianserin, and quetiapine. She had been been previously stabilized on a combination of fluoxetine and lamotrigine. Lamotrigine was discontinued when she got pregnant, but fluoxetine was continued. After the birth of a healthy girl, she soon again began to develop symptoms of depression, and the combination of lamotrigine and fluoxetine was reinstituted but with little effect.

In October 2013, she was admitted to the psychiatric ward and she fulfilled criteria for major depressive disorder. The depressive state was of a moderate severity. Lamotrigine was discontinued and fluoxetine was reduced from 80 mg daily to 60 mg daily. Quetiapine 25 mg was given for anxiolytic purposes but only on very rare occasions.

She received 9 bilateral frontotemporal treatments with ECT with 3 treatments given every week for 3 weeks (Thymatron System IV, energy level 30%). All treatments were assessed to be technically sufficient. The treatments appeared to have beneficial effect on the depression. In the course of the last ECT treatments, the spouse was concerned that her balance and her gait were seriously deteriorated and she could appear confused. Agitation was also seen intermittently. Since serotonin toxicity was suspected, fluoxetine, ECT, and quetiapine were discontinued.

Physical examination found that the patient had ataxia and generalised hyperreflexia. There was no rigidity. No hyperpyrexia, no diaphoresis, no tremor, and no myoclonus were described. Blood pressure and heart rate were in the expected range. Computerized tomography of the brain was normal.

The following days the patient was emotionally labile. She received treatment with oxazepam. The atactic gait subsided a few days after discontinuation of ECT and fluoxetine treatment. She was thereafter stabilized on a combination of nortriptyline 100 mg and melatonin. A neurologic examination 3 weeks after the start of the supposed serotonin toxicity showed no signs of ataxia or hyperreflexia.

## 3. Discussion

Existing literature reports described serotonin syndrome cases caused by SSRIs and other serotonin agonists. Reports of serotonin toxicity with ECT in combination with serotonergic agents have been sparse.

Okamoto et al. [3] reported serotonin syndrome induced by ECT and paroxetine combination and serotonin syndrome has been reported when ECT was added to clomipramine plus tryptophan treatment [4]. ECT seems to have significant effect on serotonin systems of the brain [5]. It is therefore conceivable that ECT combined with serotonin active drugs could be able to induce serotonin toxicity possibly in the form of serotonin syndrome. Some studies have found that repeated electroconvulsive shocks to animals are necessary to enhance electrophysiological and behavioural effects of serotonin, whereas treatment for a single day was without effect [6, 7]. This may be a reason why serotonin toxicity in the present case was only elicited with repeated ECT treatments.

In the reported case, the symptoms were new to the patient, and no new medication had been introduced prior to emergence of the neurologic signs. She fulfilled the criteria of serotonin syndrome as proposed by Sternbach [2]. The report of confusion and of agitation needs, however, not to be due to serotonin toxicity, since these symptoms can be secondary to ECT and to the depressive state, respectively. The normalization of the condition after discontinuation of fluoxetine and ECT support our suspicion that the symptoms had been elicited by the combination of fluoxetine and ECT, although declining concentrations of fluoxetine and norfluoxetine are expected to be present in the body long after cessation of fluoxetine administration.

## 4. Conclusion

From this and other reported cases, clinicians must be aware of the possibility of eliciting serotonin toxicity when combining serotonergic agents and ECT.
